# A Cross-Sectional Study Examining Vaccine Uptake and Attitudes Among Parents Compared to Other Adults

**DOI:** 10.1177/10901981251361433

**Published:** 2025-08-26

**Authors:** Philip M. Massey, Andrew Chuang, E. Alison Holman, Roxane Cohen Silver, Dana Rose Garfin

**Affiliations:** 1 University of California, Los Angeles, Los Angeles, CA, USA; 2 University of California, Irvine, Irvine, CA, USA

**Keywords:** vaccine hesitancy, parents, children, COVID-19, influenza

## Abstract

Encouraging vaccine uptake among U.S. residents is an increasingly important public health issue that was magnified during the COVID-19 pandemic. Vaccine hesitancy, an important correlate of vaccine uptake, has been studied extensively in parents with respect to parental attitudes and decision-making toward vaccinating their children. Less work has examined parent attitudes and behaviors regarding personal vaccine uptake and how COVID-19-related vaccine attitudes and behaviors may differ from other types of vaccine attitudes and behaviors (e.g., influenza vaccination). We surveyed a probability-based sample of 585 United States adults in November 2021. Parents (i.e., primary caregiver of at least one child aged 18 years or younger, living in the home) compared to other adults, demographics (age, sex, income, education, ethnicity, urbanicity), and political affiliation were examined as correlates of COVID-19 vaccine attitudes and COVID-19 and influenza vaccine uptake. Multivariate linear regression analyses examined attitudes toward the COVID-19 vaccine. Compared to other adults, parents of children aged 18 years or younger reported more negative attitudes toward the COVID-19 vaccine. Multivariate logistic models examined the odds of COVID-19 vaccine and influenza vaccine uptake. Compared to other adults, parents of children aged 18 years or younger had a significantly lower odds of COVID-19 vaccine uptake; differences in influenza vaccine uptake were not statistically significant. Results suggest vaccination attitudes and behaviors may be pathogen specific. Tailored public health messaging that address the concerns of caregivers may help improve vaccine uptake.

Vaccination confidence and uptake continue to be an important priority for the health, safety, and future of communities in the United States (U.S.) and around the globe. As new infectious diseases emerge and existing and historical diseases re-emerge, vaccines will remain an important tool to prevent and control the spread of disease. The COVID-19 pandemic magnified the importance of vaccination to combat viral respiratory infections, with high initial uptake: Of U.S. adults aged 18 year or older, 83.7% completed the primary COVID-19 vaccination series (Centers for Disease Control and Prevention, 2023a). Yet as of February 23, 2024, only 22.3% had received the updated bivalent booster dose (Centers for Disease Control and Prevention, 2024), indicating lower compliance as COVID-19 became endemic.

As COVID-19 vaccinations become a potential part of annual vaccine recommendations, it will be important to monitor if COVID-19 vaccine uptake is similar to other annual vaccinations such as the influenza vaccine, explore factors associated with uptake, and find areas for targeted intervention to occur. For example, while the influenza vaccine is recommended annually, during the 2022-2023 flu season, less than half (46.9%) of U.S. adults aged 18 years or older received it (Centers for Disease Control and Prevention, 2023b). This low coverage suggests the influenza vaccine may also have divergent correlates of uptake compared to the COVID-19 vaccine. Examining attitudes and norms surrounding these two vaccines (Domnich et al., 2021; Graupensperger et al., 2021; Mercadante & Law, 2021) is important to explore to promote vaccine uptake and public health more broadly.

Vaccine hesitancy is a major barrier to vaccine uptake (Bedford et al., 2018; Dubé et al., 2013; Larson et al., 2014; Olusanya et al., 2021; Schmid et al., 2017). Providing accurate, relevant, and tailored information and messaging about vaccine safety and effectiveness may be an effective way to address attitudes, historical context, and life situations that contribute to vaccine hesitancy (Olson et al., 2020). One key population often prioritized in vaccine messaging are parents (i.e., primary caregivers of children who are 18 and under and living in the home)—and for good reason, as parents are the primary decision makers for themselves and their children. While parents have been prioritized in vaccine messaging because of their role in deciding to have their children vaccinated, they are often neglected in terms of tailoring information to facilitate their own vaccination uptake (Damnjanović et al., 2018). Research has largely focused on what barriers and facilitators among parents are associated with childhood vaccine uptake. In Western countries, older parent age and higher cues to action (Davey & Gaffiero, 2024), along with recommendations from health care professionals (Davey et al., 2024), have been shown to facilitate child COVID-19 vaccination, whereas concerns about side effects and distrust in institutions serve as barriers to uptake (Davey & Gaffiero, 2024). While prior studies describe barriers to and facilitators of child vaccination, little work has focused on parents themselves, specifically how being a caregiving parent may correlate with vaccine hesitancy, vaccination intention, or vaccination behaviors (Humlum et al., 2024).

## Parental Attitudes and Acceptance Toward Child Vaccine Uptake

Studies examining parental vaccine hesitancy, especially concerning COVID-19 vaccination, have largely focused on samples of parents only (i.e., not comparing parents with other adults), with vaccine uptake among children as the main outcome (Galanis et al., 2022). Prior research demonstrated that parent COVID-19 vaccine hesitancy varies by race/ethnicity (Gray & Fisher, 2022), parent age (Davey et al., 2024), child age (Hammershaimb et al., 2022), and political affiliation (Manganello et al., 2023), among other demographic characteristics (Hudson et al., 2021). This work has produced important insights into what factors predict whether a parent will decide to have their child vaccinated, including attitudes about vaccine safety and effectiveness, trust in institutions (Davey & Gaffiero, 2024), cues to action such as a provider recommendation (Lin et al., 2021), age of the child (Galanis et al., 2022), and perceived susceptibility and severity of COVID-19 (Tang et al., 2023).

The age of the child/children has been shown to be an important factor associated with parental vaccine hesitancy, especially as it relates to COVID-19, perhaps due to the novelty of the vaccine development and the phased rollout of the vaccine to various ages over time (Assistant Secretary for Public Affairs, 2023). In general, parents of older children tend to report greater vaccination intention and uptake. For example, a study that asked parents about their COVID-19 vaccine attitudes and intentions found differences among parents, depending on the age of their children: among parents with children aged 5 to 11, 54.0% reported they were likely to vaccinate or had already vaccinated their child, compared to 69.7% of parents with children aged 12 to 17 (Hammershaimb et al., 2022). The lower rates of vaccination among children aged 5 to 11 have been shown in other studies among Western countries, with only 46.4% of parents intending to vaccinate against COVID-19 (Davey & Gaffiero, 2024). Similarly, when examining vaccination status among parents themselves, uptake was higher for parents with 12 to 17-year-olds (78.6%) than parents with 5 to 11-year-olds (50.7%) (Harris et al., 2023). These patterns suggest that vaccine uptake, particularly with a new vaccine (i.e., a new technology such as mRNA that was used in many COVID-19 vaccines), may diffuse differently based on group characteristics.

The diffusion of innovations theory posits that new behaviors (e.g., deciding to get a new vaccine) may be adopted at different rates among populations based on various factors and group characteristics. The theory identifies various components of the innovation that influence adoption, including relative advantage, complexity, compatibility, and trialability (Dearing & Singhal, 2020). Prior studies have used diffusion of innovations to better understand vaccine uptake from individual to system levels, including studies on the readiness of COVID-19 vaccine uptake (Mo et al., 2021), the role of school nurses in human papilloma virus (HPV) vaccine uptake (Rosen & Goodson, 2013), and the diffusion of pharmacy-based influenza vaccination (Chun et al., 2016). The health belief model adds an additional layer of understanding to diffusion of innovations by introducing concepts related to perceived benefits, risks of a behavior, and cues to action (Rosenstock, 2000). Prior studies have shown how higher cues to action (Davey et al., 2024), including a health care provider recommendation (Fisher et al., 2023), predict higher vaccine intention and uptake. Taken together, these theories offer potential insights into why new technologies and behaviors (i.e., deciding to receive a new vaccine) may be adopted differently among different groups. When comparing the COVID-19 and influenza vaccines, parents may demonstrate varying attitudes and behaviors toward the vaccines given the more recent introduction of the COVID-19 vaccine. If differences in vaccine uptake are present in parents compared to other adults, tailored and targeted information addressing the various components of the innovation may help facilitate adoption, for example, using pediatrician appointments to concurrently promote adult vaccination for parents.

These family-level factors occur within a broader social context. Importantly, vaccine hesitancy and decision-making has grown increasingly divided along partisan lines (Relihan et al., 2023). In a nationally representative sample of 6,514 U.S. residents, identifying as a Republican compared to a Democrat was positively associated with vaccine hesitancy and negatively associated with intention to vaccinate (Relihan et al., 2023). This finding was echoed in analyses of county-level data, which showed lower COVID-19 vaccination rates in counties with a higher percentage of ballots cast for the Republican presidential nominee in the 2020 election (Albrecht, 2022). These divisions extend to decisions of caregivers to vaccinate their children: in a distinct nationally representative sample of parents, Republican or Republican- leaning parents were more likely to refuse to vaccinate their children (Panchalingam & Shi, 2022). Given these findings, it is also important to account for the potential effect of political identity on vaccine hesitancy and vaccination behaviors.

## The Present Study

Here, we address the gaps in the extant literature by exploring and comparing vaccine attitudes and behaviors among “parents” (defined here as caregivers of children currently living at home who are aged 18 years and younger, consistent with the age most U.S. children graduate high school) with other adults (defined here as those without children or whose children were 19 and older or no longer living at home). Specifically, we seek to examine how parent status may differentially correlate with COVID-19-related vaccine hesitancy and COVID-19 vaccination uptake. We also include vaccine uptake for a less-politicized viral pathogen and more familiar vaccination, influenza, to test a potential spillover effect between COVID-19-related vaccine hesitancy and other vaccinations that are recommended annually. We had several hypotheses.

**Hypothesis 1:** Attitudes toward COVID-19 vaccination will differ among parents with children aged 18 years or younger compared with other adults.
**Hypothesis 2:** Behavioral intentions toward a) COVID-19 vaccination and b) influenza vaccination will differ among parents with children aged 18 years or younger when compared to other adults.
**Hypothesis 3:** Individual-level factors, including political party identification and age of child, will be associated with vaccine uptake.

## Methods

### Study Overview

Participants were drawn from an ongoing, longitudinal study of a representative, probability-based sample of U.S. residents using the NORC AmeriSpeak Panel. A subsample of participants who provided data on their parental status were included in the present analyses. Parental status, political party identification, and demographic indicators (i.e., age, race/ethnicity, education, gender, income, and urban/rural residence) were examined as predictors of COVID-19 vaccine attitudes and COVID-19 and influenza vaccination behavior.

### Sample and Procedure

The NORC AmeriSpeak Panel is a probability-based panel of 35,000 U.S. households. NORC’s AmeriSpeak Panel randomly selected participants from their panel to form a representative sample of U.S. households. Sample stratification promoted representativeness for age, gender, race/ethnicity, and education. The Wave 1 survey was fielded to a sample of 11,139 panelists in three consecutive 10-day cohorts from 3/18/2020 (five days after the U.S. declaration of a national emergency due to COVID-19) to 4/18/2020; 6,514 responded (58.5% completion rate). In each cohort, most respondents (>85%%) completed the survey in the first three days of data collection. Participants received an email stating that the 20-minute survey was available; they completed the self-administered, confidential survey online. Details of the initial study design have been detailed elsewhere (Holman et al., 2020). A second wave of data collection occurred in September-October 2020, although data from that wave are not included in this report. A follow-up survey (Wave 3) was fielded 11/8/2021-11/24/2021; 6,486 Wave 1 panelists were eligible and 4,881 responded (75.3% completion rate). NORC compensated AmeriSpeak panelists with points worth a cash equivalent of $4-$10 at each wave. Participants provided informed consent when they joined the NORC panel and were informed that their identities would remain confidential. All procedures for this study were approved by the Institutional Review Board at the University of California, Irvine.

### Subsample for Present Analyses

NORC regularly updates information on panelists’ demographics, including number of persons in the home. Between 2/3/2022 and 4/21/2022, for participants indicating >1 person lived in their household, primary caregiver/parental status was ascertained by asking if the panelist was the parent or guardian of a child (including adopted child, stepchild, grandchild, or foster child) living in the home. Age of the child/children was also assessed. Reliable data from 814 panelists were obtained. Data were deemed “unreliable” if observations were contradictory or missing either the age of the child/children or the respondent’s relationship to them (e.g., respondent status as a parent or guardian of the child was not provided, age of child/children not provided). Those with reliable data tended to be younger (OR=0.97, 95% CI, 0.96, 0.98, *p*<.001), lower income (OR=0.94, 95% CI, 0.89, 0.99, *p*=.012), and from an urban, compared to rural, community (OR=1.46, 95% CI, 1.22, 1.76, *p*<.001).

From this subsample, two categories of respondents were created for our analyses: *parents* (which includes all primary caregivers—including biological, foster, and step parents as well as grandparents—to children aged 18 and under currently living in the home) and *other adults* (including those without children or whose children were 19 and older or no longer living at home). In preliminary analyses, parents of children ≤18 were significantly younger, on average, than other adults (*p*<.05), reflecting that most adults older than middle age are no longer primary caregivers to children aged 18 and under living in the home. To control for potential age differences between parents and other adults that could be associated with outcomes, we restricted our sample to only included panelists who, as of 2022, were < 65 years old (based on general definitions of “middle age”) (Feig et al., 2021; Lachman et al., 2015; Livingston et al., 2020). The oldest parent was 58 years old prior to this decision rule. We were left with a final subsample of 586 individuals that comprise the analyses presented in this report.

## Measures

### Independent Variables

#### Parent Status

We created a dichotomous variable: “other adult” (*does not have a child aged 18 years or younger living at home, for whom they are a primary caregiver*) = 0, “parent” (*primary caregiver to at least one child aged 18 years or younger living at home)* = 1.

#### Political Party Identification

Participants reported their political party affiliation, which was updated prior to Wave 3 data collection on a seven-item scale: 1=*strong Democrat*, 2=*moderate Democrat*, 3=*lean Democrat*, 4=*don’t lean/independent/none*, 5=*lean Republican*, 6=*moderate Republican*, 7=*strong Republican*.

#### Demographics

NORC collects demographic information including age, race/ethnicity, education, gender, income, and urban/rural residence when participants enroll into the AmeriSpeak panel; these variables are updated annually for accuracy.

### Dependent Variables

#### COVID-19 Vaccine Attitudes

At Wave 3, attitudes about the COVID-19 vaccine were assessed via a nine-item measure (endpoints: 1=*strongly disagree* to 5=*strongly agree*): (1) COVID-19 vaccines help me protect my loved ones; (2) COVID-19 vaccines are an effective way to end the pandemic, (3) Everyone who is eligible and medically able should get a COVID-19 vaccine, (4) COVID-19 vaccinations are safe; (5) COVID-19 vaccines help me protect my community; (6) COVID-19 vaccinations conflict with my religious views; (7) COVID-19 vaccines were developed too quickly; (8) It is better to build immunity naturally than to get a vaccine; and (9) COVID-19 vaccine mandates infringe on civil liberties. In the survey, positive (items 1–5) and negative (items 6–9) were alternated; negative items were reversed scored. Items were then averaged; higher scores indicate more positive attitudes about the COVID-19 vaccine (*α* = .95).

### Vaccination Behavior

At Wave 3, vaccination intentions and behaviors were assessed with the following items for both the COVID-19 vaccine and the annual influenza vaccine: “Have you gotten the COVID-19 vaccine?” with response options 1) yes, voluntarily, 2) yes, but only because I was required to by my employer/school, 3) no, but I plan to, 4) no, and I do not plan to, and 5) no, I am medically unable. Items were coded into a dichotomous variable: Yes, vaccinated (combining responses 1 and 2 above); No, not vaccinated (combining responses 3, 4, and 5 above). These groupings were informed by conceptual and statistical considerations, including small sample sizes for responses 2 (*n* = 17, 2.92%) and 5 (*n* = 7, 1.20%). An analogous item was used to assess influenza vaccine behavior.

### Analytic Strategy

First, descriptive statistics were calculated to identify any socio-demographic differences between parents of children ≤18 and other adults, using *t*-tests for continuous variables and χ^2^ for categorical variables.

Next, the associations between key predictor variables and COVID-19 vaccine attitudes were examined using a series of ordinary least squares regression models. Variables were entered using a hierarchical variable entry strategy to illustrate the relative contributions of our a-priori, conceptually derived independent variables. Variables were entered in theoretically meaningful blocks: demographics (including respondent gender, age, urbanicity) and parental status were entered first (Model 1) and political party identification was entered second (Model 2). Using an analogous approach, correlates of COVID-19 vaccination behavior and influenza vaccination behavior were examined using a series of two multivariate logistic regression analyses. Interaction terms between caregiver status and gender, political ideology, and urbanicity were calculated and analyzed in additional exploratory models for each dependent variable (i.e., COVID-19 vaccine attitudes, COVID-19 vaccination behavior, and influenza vaccination behavior). To ascertain if the age of the child/children were associated with outcomes, analyses were replicated dividing the parent group into two categories based on the age of the oldest child (parent to child/children <12 years old; parent to child/children 12–18 years old), consistent with the rollout of the initial COVID-19 vaccine approval for children in 2021. All study materials are available on the Inter-university Consortium for Political and Social Research website (https://www.icpsr.umich.edu/web/ICPSR/studies/39032/summary).

## Results

Table 1 presents the description of the sample and bivariate (unadjusted) between-group differences between parents and other adults. In general, parents tended to be more educated (*X*^ 2^[1] = 7.05, *p* = .007) and earn higher incomes (*X*^2^ [3] = 18.88, *p* < .001). In addition, a lower proportion of parents had received the COVID-19 vaccine (*X*^2^[1] = 14.81, *p* < .001). There were no differences between parents of children ≤18 years old and other adults in political party identification, race/ethnicity, gender, urbanicity, or influenza vaccine uptake. Table 2 presents the results examining Hypothesis 1: *Attitudes toward COVID-19 vaccination will differ among parents with children aged 18 years or younger compared with other adults*. After controlling for demographics and political party identification, we found that parents of children ≤18 years old reported, on average, more negative COVID-19 vaccine attitudes compared to other adults (the reference group) (β = −0.16, *p* < .001). Participants who reported stronger Republican views (as compared to stronger Democrat views) also had, on average, more negative COVID-19 vaccine attitudes (β = −0.50, *p* < .001). Conversely, having a Bachelor’s degree or higher was significantly associated with more positive COVID-19 vaccine attitudes (β = 0.20, *p* < .001).

**Table 1:** Description of the Sample, N = 585.

	Total	Parent of child/children ≤18 years old^a^	Other adult^b^		Test of between-group differences^c^	*df*	Effect size (Cohen’s *d* or Cramer’s *V*)
Variable	*N*=585 (100%)	*n*=333 (56.9%)	*n*=252 (43.1%)	*p* value			
Age, mean (sd)	40.9 (10.9)	37.57 (7.3)	45.33 (13.1)	<0.001	9.18	583	0.87
Gender identification							
Male	222 (37.9%)	122 (36.6%)	100 (39.7%)	0 .452	0.13	1	0.02
Female	363 (62.1%)	211 (63.4%)	152 (60.3%)				
Race/Ethnicity							
White, non-Hispanic	424 (72.5%)	245 (73.6%)	179 (71.0%)	0.351	0.66	3	0.03
Black, non-Hispanic	45 (7.7%)	27 (8.1%)	18 (7.1%)				
Other/2+races, non-Hispanic	65 (11.1%)	38 (11.4%)	27 (10.7%)				
Hispanic	51 (8.7%)	23 (6.9%)	28 (11.1%)				
Education							
Less than a bachelor’s degree	299 (51.1%)	154 (46.2%)	145 (57.5%)	0.007	7.05	1	0.11
Bachelor’s degree or higher	286 (48.9%)	179 (53.8%)	107 (42.5%)				
Urbanicity of residence							
Nonurban	143 (24.4%)	87 (26.1%)	56 (22.2%)	0.277	1.24	1	−0.05
Urban	442 (75.6%)	246 (73.9%)	196 (77.8%)				
4-level household income							
Less than $30,000	124 (21.2%)	56 (16.8%)	68 (27.0%)	<.001	18.88	3	0.18
$30,000 to under $60,000	165 (28.2%)	85 (25.5%)	80 (31.7%)				
$60,000 to under $100,000	166 (28.4%)	101 (30.3%)	65 (25.8%)				
$100,000 or more	130 (22.2%)	91 (27.3%)	39 (15.5%)				
Political party identification^d^, mean (sd)	3.8 (2.0)	3.9 (2.0)	3.6 (2.1)	0.054	0.13	583	0.02
COVID-19 vaccine attitudes^e^, mean (sd)	3.6 (1.2)	3.4 (1.2)	3.8 (1.1)	<0.001	4.46	583	0.37
COVID vaccine behavior							
No, not vaccinated for COVID-19	144 (24.8%)	102 (30.8%)	42 (16.9%)	<0.001	14.81	1	−0.16
Yes, vaccinated for COVID-19	436 (75.2%)	229 (69.2%)	207 (83.1%)				
Influenza vaccine behavior							
No, not vaccinated for seasonal influenza	359 (61.6%)	209 (63.0%)	150 (59.8%)	0.433	0.62	1	−0.03
Yes, vaccinated for seasonal influenza	224 (38.4%)	123 (37.0%)	101 (40.2%)				

a Parents defined as primary caregiver to child/children aged 18 years or younger who were living at home; ^b^Any adult who is not currently a primary caregiver to a child aged 18 and younger who is living at home; ^c^*t*-tests used for continuous variables, *X*^2^ used for categorical variables, df = degrees of freedom; ^d^1 = *Strong Democrat*; 7 = *Strong Republican*; ^e^Range from 1–5 where higher numbers correspond to more positive attitudes.

**Table 2:** Multivariate Ordinary Least Squares Regressions of the Relationship between Parental Status^a^, Key Covariates, and COVID-19 Vaccination Attitudes (N = 585).

	Model 1				Model 2			
Variable	β	b	95% CI	*p* value	β	b	95% CI	*p* value
Age	0.01	0.001	−0.01, 0.01	0.798	0.07	0.01	−0.0002, 0.02	0.055
Gender identification								
Male (ref)	-	-	-	-	-	-	-	-
Female	0.02	0.05	−0.14, 0.23	0.633	−0.03	−0.07	−0.23, 0.09	0.389
Education								
Less than a bachelor’s degree (ref)	-	-	-	-	-	-	-	-
Bachelor’s degree or higher	0.28	0.67	0.47, 0.86	<.001	0.20	0.47	0.30, 0.64	<0.001
Race/Ethnicity								
White, non-Hispanic (ref)	-	-	-	-	-	-	-	-
Black, non-Hispanic	0.04	0.17	−0.18, 0.51	0.340	−0.09	−0.39	−0.69, −0.09	0.012
Other/2+races, non-Hispanic	0.04	0.17	−0.12, 0.46	0.253	0.01	0.02	−0.22, 0.27	0.849
Hispanic	0.07	0.29	−0.04, 0.61	0.082	0.01	0.03	−0.25, 0.31	0.839
Household income	0.18	0.12	0.06, 0.18	<0.001	0.13	0.09	0.04, 0.14	<0.001
Parental status^a^								
Other adult (ref)	-	-	-	-	-	-	-	-
Parent to child/children ≤18	−0.24	−0.58	−0.78, −0.38	<0.001	−0.16	−0.38	−0.56, −0.21	<0.001
Urbanicity of residence								
Nonurban (ref)	-	-	-	-	-	-	-	-
Urban					0.07	0.20	0.02, 0.38	0.034
Political party identification^b^					−0.50	−0.29	−0.33, −0.25	<0.001
Intercept		2.92	2.34, 3.30	<0.001		3.88	3.44, 4.33	<0.001
R-squared	0.18				0.41			

*Note.* CI=confidence interval; Ref=reference group coded 0; ^a^Parents defined as primary caregiver to child/children aged 18 years and younger who were living at home; ^b^1=*Strong Democrat* to 7=*Strong Republican*.

Table 3 presents results testing Hypothesis 2: *Behavioral intentions toward a) COVID-19 vaccination and b) influenza vaccination will differ among parents with children aged 18 years or younger compared with other adults*. Compared to other adults, parents of children ≤18 years old reported lower odds of COVID-19 vaccine uptake (Odds Ratio [OR] = 0.43, *p* < .001). Similarly, those identifying more strongly as Republicans compared to Democrats reported lower odds of COVID-19 vaccine uptake (OR = 0.66, *p* < .001). In contrast, respondent age (OR = 1.03, *p* = .006), education level (OR = 2.83, *p* < .001), and income (OR = 1.33, *p* < .001) were positively, significantly associated with higher odds of COVID-19 vaccine uptake.

**Table 3.** Multivariate Logistic Regressions of the Relationships Between Parental Status^a^, Key Covariates, and COVID-19 Vaccination Behavior (N = 580).

	Model 1				Model 2			
Variable	OR	95% CI	*SE*	*p* value	OR	*SE*	95% CI	*p* value
Age	1.02	1.00, 1.04	0.01	0.053	1.03	0.01	1.01, 1.06	0.006
Gender identification								
Male (ref)	-	-		-	-		-	-
Female	1.03	0.67, 1.59	0.23	0.898	0.89	0.21	0.56, 1.41	0.605
Education								
Less than a bachelor’s degree (ref)	-	-		-	-		-	-
Bachelor’s degree or higher	3.34	2.08, 5.35	0.80	<.001	2.83	0.72	1.71, 4.66	<.001
Race/Ethnicity								
White, Non-Hispanic (ref)	-	-		-	-		-	-
Black, Non-Hispanic	0.95	0.46, 1.97	0.35	0.886	0.37	0.16	0.16, 0.84	0.018
Other/2+races, Non-Hispanic	1.41	0.68, 2.93	0.53	0.360	1.17	0.47	0.53, 2.56	0.696
Hispanic	1.45	0.68, 3.09	0.56	0.332	0.91	0.38	0.40, 2.04	0.812
Household income	1.34	1.16, 1.56	0.10	<.001	1.33	0.11	1.14, 1.56	<.001
Parental status^a^								
Other adult (ref)	-	-		-	-		-	-
Parent to child/children ≤18	0.37	0.23, 0.60	0.09	<.001	0.43	0.11	0.26, 0.72	0.001
Urbanicity of residence								
Nonurban (ref)	-	-		-	-		-	-
Urbanicity					2.02	0.50	1.25, 3.27	0.004
Political party identification^b^					0.66	0.04	0.58, 0.75	<.001
Intercept	0.43	0.14, 1.29	0.24	0.133	1.24	0.82	0.34, 4.52	0.742
χ^2^, Log likelihood		95.90, −277.10			154.83, −247.64			

Notes: OR=Odds Ratio; CI=Confidence Interval; Ref=reference group coded 0; ^a^Parents defined as primary caregiver to child/children aged 18 years or younger who were living at home; ^b^1=*Strong Democrat* to 7=*Strong Republican*.

Table 4 presents results examining Hypothesis 3: *Individual-level factors, including political party identification and age of child, are associated with vaccine uptake*. Parents of children ≤18 years old, urbanicity, and income were not statistically significant correlates of influenza vaccine uptake. Identifying more strongly as a Republican, compared to a Democrat, was associated with lower odds of vaccine uptake (OR = 0.84, *p* < .001). In contrast, older age (OR = 1.02, *p* = .007) and having a bachelor’s degree or higher (OR = 1.79, *p* = .003) were significantly associated with a higher likelihood of influenza vaccine uptake.

**Table 4.** Multivariate Logistic Regressions of the Relationship Between Parental Status^a^, Key Covariates, and Influenza Vaccination Behavior (N = 583).

	Model 1				Model 2			
Variable	OR	*SE*	95% CI	*p* value	OR	*SE*	95% CI	*p* value
Age	1.02	0.01	1.00, 1.04	0.022	1.02	0.01	1.01, 1.04	0.007
Gender identification								
Male (ref)	-		-	-	-		-	-
Female	1.49	0.28	1.03, 2.14	0.032	1.40	0.27	0.97, 2.03	0.072
Education								
Less than a bachelor’s degree (ref)	-		-	-	-		-	-
Bachelor’s degree or higher	1.96	0.38	1.34, 2.86	0.000	1.79	0.35	1.22, 2.64	0.003
Race/Ethnicity								
White, non-Hispanic (ref)	-		-	-	-		-	-
Black, non-Hispanic	1.22	0.41	0.63, 2.38	0.552	0.89	0.31	0.45, 1.77	0.734
Other/2+races, non-Hispanic	0.69	0.20	0.39, 1.22	0.199	0.63	0.19	0.35, 1.13	0.122
Hispanic	1.10	0.35	0.59, 2.06	0.763	0.98	0.32	0.51, 1.86	0.939
Household income	1.10	0.06	0.99, 1.23	0.081	1.09	0.06	0.97, 1.22	0.145
Parental status^a^								
Other adult (ref)	-		-	-	-		-	-
Parent to child/children ≤18	0.87	0.17	0.59, 1.28	0.492	0.97	0.20	0.66, 1.45	0.898
Urbanicity of residence								
Nonurban (ref)	-		-	-	-		-	-
Urban					0.97	0.21	0.64, 1.49	0.902
Political party identification^b^					0.84	0.04	0.77, 0.92	<0.001
Intercept	0.11	0.05	0.04, 0.28	<0.001	0.21	0.11	0.07, 0.58	0.003
χ^2^, log likelihood		33.01, −371.83				48.05, −364.30		

Notes: OR=Odds Ratio; CI=Confidence Interval; Ref=reference group coded 0; ^a^Parents defined as primary caregiver to child/children aged 18 years or younger who were living at home; ^b^1=*Strong Democrat* to 7=*Strong Republican*.

There were no significant differences in vaccine uptake according to the age of the oldest child (i.e., parents of child/children <12 years old compared to parents of child/children 12 to 18 years old). Analyses were analogous to those with the parent group combined (i.e., parents of children ≤18 years old compared to other adults). See Supplemental Tables A1–A3 for full results. No interaction terms were statistically significant in any models for any of the dependent variables (all *p* values >.05).

## Discussion

This study sought to examine how attitudes and behavioral intentions related to the COVID-19 and influenza vaccines varied among parents with children aged 18 years or younger, compared with other adults. Findings from our study demonstrate that parents of children ≤18 years old may have vaccine attitudes and uptake behaviors that are different from other adults. Our results found that compared to other adults, parents with children ≤18 years old had more negative COVID-19 vaccine attitudes as well as lower odds of getting the COVID-19 vaccine for themselves. There were no differential relationships found depending on the age of the child/children at home. However, these patterns did not hold true when considering the influenza vaccine—parents with children ≤18 years old showed no differences in influenza vaccine uptake when compared to other adults. Further, while initial COVID-19 vaccine uptake was generally high among all adults in our sample (75.2%), influenza vaccine uptake was relatively lower (38.4%).

Results highlight that parent and caregiver role modeling matters. Parents with children living at home who are 18 and under often model healthy practices and behaviors. The impact of parent role modeling and social norms relative to the COVID-19 vaccine is evidenced by research showing that parent norms toward the vaccine predicted adolescent willingness to receive the vaccine—the greater the perceived support for the COVID-19 vaccine by one’s parents, the higher the adolescents’ intentions were to vaccinate (Rogers et al., 2021). Behavior change theories that highlight the importance of social norms, observational learning, and reciprocal determinism, such as the Social Cognitive Theory, will remain important in designing and tailoring programs and messages that speak to the impact of parent role modeling (Islam et al., 2023). Indeed, these can highlight the importance of “cues to action” when making modeling vaccine decisions in the family context. With the influenza vaccination already recommended annually (Grohskopf et al., 2021), and the potential for COVID-19 vaccinations to be recommended annually (Bar-On et al., 2022), understanding vaccine attitudes and uptake among parents with children ≤18 years old is important not only for parents themselves but for their children as well.

We examined several interaction terms with parental status in our study and found no moderating effects. For example, compared with other adults, parents with children ≤18 years old held more negative attitudes and had lower odds of getting the COVID-19 vaccine regardless of urbanicity or political affiliation. Similarly, we did not find a significant interaction effect with gender or race/ethnicity variables, deviating from prior work that demonstrated that mothers and Black parents were more vaccine hesitant compared to mothers and parents identifying as White (Simonson et al., 2021). Finally, while parents with children ≤18 years old overall were more COVID-19 vaccine hesitant when compared to other adults, we did not find differences in COVID-19 vaccine attitudes or vaccine uptake among parents depending on the age of their children. More specifically, we did not find significant differences in vaccine attitudes or uptake between parents of children <12 years old and parents of children 12 to 18 years old. This finding departs from prior research that has shown differences between these two categories of caregivers (Funk, 2017).

Our findings support the need to prioritize strategies that address vaccine hesitancy among parents and caregivers with children living at home who are 18 and under (Limaye et al., 2021). Strategies that have been identified in other research include tailoring messaging to increase the salience of vaccines—a strategy that draws from the diffusion of innovations theory and may be particularly relevant to parents when making decisions relative to newer vaccines (i.e., COVID-19) as compared to more familiar vaccines (i.e., influenza) (Panozzo et al., 2020). This may be particularly important when considering newer or emerging vaccines as was witnessed with the COVID-19 pandemic. Emerging mRNA technology, compared to conventional vaccinations, was associated with higher vaccine hesitancy (Leong et al., 2022). Further, strong and personalized provider recommendations, an example of a cue to action as described in the Health Belief Model, have been shown to have a positive impact on vaccine acceptance among vaccine hesitant parents (Limaye et al., 2021). In the context of our findings, additional research may be needed to explore the impact of provider recommendations not only on child vaccine uptake, but also the impact they may have on parent vaccine uptake and household vaccine uptake. Parents model behaviors to children and strengthening vaccine uptake among the parents may have spillover effects that help increase vaccine uptake among families and households (Humlum et al., 2024).

### Strengths and Limitations

Strengths of our study include a probability-based sampling design, which allows for increased generalizability, particularly with respect to underrepresented groups including rural areas that frequently exhibit undercoverage in survey studies. More specifically, opt-in panels, which defined much of the previous research on COVID-19 (Pierce et al., 2020), frequently suffer from substantial selection biases, typically oversampling volunteers who are engaged and interested in the topic and who have internet access (Callegaro et al., 2014; Yeager et al., 2011), leaving out underrepresented groups including those from rural areas (Perrin & Atske, 2021).

While our study offers numerous strengths, there are several limitations worth noting. First, we did not have parent/caregiver status for our complete sample of 6,514, and some key covariates were associated with missing data. However, recruitment for the AmeriSpeak panel still relies on probability-based methods; thus, our sample provides more representativeness and generalizability than traditional opt-in survey studies. We did not ask about general vaccine attitudes, or specific attitudes toward the influenza vaccine. Thus, we could not compare patterns of vaccine hesitancy between COVID-19, influenza, other viral threats. Further, we specifically focused on parents in our sample who had children 18 and under living at home; there were likely parents or former caregivers of children in our “other adult” category, who had children who were no longer living at home or who may have been living at home due to the pandemic or other reasons (e.g., financial constraints, attending college locally). While these children were likely no longer under their caregiver’s medical decision-making, being a former caregiver or biological parent may have impacted outcomes. We relied on self-report for vaccine uptake. Finally, our variables were not assessed at repeated timepoints, thus precluding our ability to make causal inferences over time.

Parents and caregivers have been priority audiences for vaccine campaigns to encourage them to vaccinate their children. As the current political climate becomes increasingly skeptical about established vaccine science, parent concerns about vaccine safety may increase accordingly, along with increased vaccine hesitancy and reduced vaccination rates. Indeed, recent years have seen an erosion in vaccine confidence and coverage, which may increase without clinical, public health, and media-based interventions (Eagan et al., 2023). It is imperative for future research to focus on effective public health and clinical communication strategies that compassionately, accurately, and effectively address parents’ concerns.

Our study showed that parents with children 18 and under living at home should themselves be priority audiences for vaccine campaigns, as they demonstrated more negative vaccine attitudes and lower vaccine uptake compared to other adults. More research is needed to further uncover why these differences exist, and if vaccine hesitancy that may originate with safety concerns regarding one’s children may spill over into parent’s attitudes and vaccine uptake for themselves. Programs and campaigns that seek to strengthen vaccine uptake among children by engaging with parents must also consider parent vaccine uptake as a unique outcome. This approach has the potential to contribute to innovations in family- and household-level vaccine uptake models.

## Supplemental Material

sj-docx-1-heb-10.1177_10901981251361433 – Supplemental material for A Cross-Sectional Study Examining Vaccine Uptake and Attitudes Among Parents Compared to Other Adults Supplemental material, sj-docx-1-heb-10.1177_10901981251361433 for A Cross-Sectional Study Examining Vaccine Uptake and Attitudes Among Parents Compared to Other Adults by Philip M. Massey, Andrew Chuang, E. Alison Holman, Roxane Cohen Silver and Dana Rose Garfin in Health Education & Behavior
