# Supplementary material for: A Cross-Sectional Study Examining Vaccine Uptake and Attitudes Among Parents Compared to Other Adults
Source: Health Educ Behav. 2025 Aug 26;53(2):128–38. doi: 10.1177/10901981251361433 (PMC12953656; doi:10.1177/10901981251361433)
Supplement: sj-docx-1-heb-10.1177_10901981251361433 – Supplemental material for A Cross-Sectional Study Examining Vaccine Uptake and Attitudes Among Parents Compared to Other Adults [file sj-docx-1-heb-10.1177_10901981251361433.docx]

**A Cross-Sectional Study Examining Vaccine Uptake and Attitudes Among Parents Compared to Other Adults**

**Supplemental File**

Table A1. Multivariate ordinary least squares regressions of the relationship between parental status^a^ (including age of child/children), key covariates, and COVID-19 vaccination attitudes^a^ (N=585)

|  | Model 1 | | | | Model 2 | | | |
| --- | --- | --- | --- | --- | --- | --- | --- | --- |
| Variable | β | b | 95% CI | *p*-value | β | b | 95% CI | *p*-value |
| Age | 0.03 | 0.003 | -0.01, 0.01 | 0.453 | 0.08 | 0.01 | 0.001, 0.02 | 0.033 |
| Gender identification |  |  |  |  |  |  |  |  |
| Male (ref) | - | - | - | - | - | - | - | - |
| Female | 0.02 | 0.04 | -0.15, 0.23 | 0.670 | -0.03 | -0.07 | -0.23, 0.09 | 0.380 |
| Education |  |  |  |  |  |  |  |  |
| Less than a bachelor’s degree (ref) | - | - | - | - | - | - | - | - |
| Bachelor’s degree or higher | 0.28 | 0.65 | 0.46, 0.85 | <.001 | 0.19 | 0.47 | 0.30, 0.64 | <.001 |
| Race/Ethnicity |  |  |  |  |  |  |  |  |
| White, non-Hispanic (ref) | - | - | - | - | - | - | - | - |
| Black, non-Hispanic | 0.04 | 0.18 | -0.16, 0.53 | 0.291 | -0.08 | -0.37 | -0.67, -0.07 | 0.015 |
| Other/2+races, non-Hispanic | 0.04 | 0.16 | -0.12, 0.45 | 0.262 | 0.01 | 0.02 | -0.22, 0.27 | 0.851 |
| Hispanic | 0.07 | 0.28 | -0.04, 0.60 | 0.088 | 0.01 | 0.03 | -0.25, 0.31 | 0.845 |
| Household income | 0.18 | 0.12 | 0.06, 0.18 | <0.001 | 0.13 | 0.09 | 0.04, 0.14 | <0.001 |
| Parental status^a^ |  |  |  |  |  |  |  |  |
| Other adult (ref) | - | - | - | - | - | - | - | - |
| Parent, child/children <12 years old | -0.20 | -0.4 | -0.69, -0.26 | <0.001 | -0.14 | -0.34 | -0.52, -0.15 | <0.001 |
| Parent, child/children 12-18 years old | -0.24 | -0.83 | -1.11, -0.55 | <0.001 | -0.15 | -0.50 | -0.74, -0.26 | <0.001 |
| Urbanicity of residence |  |  |  |  |  |  |  |  |
| Non-urban (ref) | - | - | - | - | - | - | - | - |
| Urban |  |  |  |  | 0.07 | 0.20 | 0.01, 0.38 | 0.035 |
| Political party identification^b^ |  |  |  |  | -0.49 | -0.29 | -0.33, -0.25 | <.001 |
| Intercept |  | 2.82 | 2.34, 3.30 | <0.001 |  | 3.83 | 3.37, 4.28 | <.001 |
| R-squared | 0.18 |  |  |  | 0.41 |  |  |  |
| Notes: CI = confidence interval. Ref=reference group coded 0; ^a^Parents defined as primary caregiver to child/children age18 and younger who were living at home; ^b^1=*Strong Democrat* to 7=*Strong Republican*. | | | | | | | | |

### Table A2. Multivariate logistic regressions of the relationship between parent category^a^ (including age of child/children), key covariates, and COVID-19 vaccination behavior (*N*=580)

|  | Model 1 | | | Model 2 | | |
| --- | --- | --- | --- | --- | --- | --- |
| Variable | OR | 95% CI | *p*-value | OR | 95% CI | *p*-value |
| Age | 1.02 | 1.00, 1.05 | 0.046 | 1.03 | 1.01, 1.06 | 0.008 |
| Gender Identification |  |  |  |  |  |  |
| Male (reference group) | - | - | - | - | - | - |
| Female | 1.03 | 0.66, 1.59 | 0.909 | 0.89 | 0.56, 1.41 | 0.608 |
| Education |  |  |  |  |  |  |
| Less than a bachelor’s degree (ref) | - | - | - | - | - | - |
| Bachelor’s degree or higher | 3.30 | 2.06, 5.30 | <0.001 | 2.84 | 1.71, 4.70 | <0.001 |
| Race/Ethnicity |  |  |  |  |  |  |
| White, non-Hispanic (ref) | - | - | - | - | - | - |
| Black, non-Hispanic | 0.96 | 0.46, 1.99 | 0.911 | 0.37 | 0.16, 0.84 | 0.018 |
| Other/2+races, non-Hispanic | 1.41 | 0.68, 2.93 | 0.361 | 1.17 | 0.53, 2.56 | 0.696 |
| Hispanic | 1.45 | 0.68, 3.10 | 0.331 | 0.91 | 0.40, 2.04 | 0.809 |
| Household income | 1.34 | 1.16, 1.56 | <.001 | 1.33 | 1.14, 1.56 | <0.001 |
| Parent category^a^ |  |  |  |  |  |  |
| Other adult (ref) | - | - | - | - | - | - |
| Parent, child/children < 12 years old | 0.39 | 0.23, 0.65 | <0.001 | 0.48 | 0.25, 0.75 | 0.003 |
| Parent, child/children 12-18 years old | 0.33 | 0.18, 0.63 | 0.001 | 0.45 | 0.23, 0.87 | 0.019 |
| Urbanicity of residence |  |  |  |  |  |  |
| Non-urban (ref) | - | - | - | - | - | - |
| Urbanicity |  |  |  | 2.02 | 1.25, 3.27 | 0.004 |
| Political party identification^b^ |  |  |  | 0.66 | 0.58, 0.75 | <0.001 |
| Intercept | 0.41 | 0.14, 1.25 | 0.117 | 1.26 | 0.34, 4.72 | 0.728 |
| χ², Log Likelihood | 96.13, -276.98 | | | 154.85, -247.63 | | |
| Notes: OR=Odds Ratio; CI=Confidence Interval. CI = confidence interval. Ref=reference group coded 0; ^a^Parents defined as primary caregiver to child/children age18 and younger who were living at home; ^b^1=*Strong Democrat* to 7=*Strong Republican*. | | | | | | |

### Table A3. Multivariate logistic regressions of the relationship between parent category^a^ (including age of the child/children),^a^ key covariates, and influenza vaccination behavior (*N* = 583)

|  | Model 1 | | | Model 2 | | |
| --- | --- | --- | --- | --- | --- | --- |
| Variable | OR | 95% CI | *p*-value | OR | 95% CI | *p*-value |
| Age | 1.02 | 1.01, 1.04 | 0.011 | 1.03 | 1.01, 1.05 | 0.004 |
| Gender identification |  |  |  |  |  |  |
| Male (ref) | - | - | - | - | - | - |
| Female | 1.48 | 1.03, 2.13 | 0.036 | 1.40 | 0.97, 2.03 | 0.074 |
| Education |  |  |  |  |  |  |
| Less than a bachelor’s degree (ref) | - | - | - | - | - | - |
| Bachelor’s degree or higher | 1.93 | 1.32, 2.81 | 0.001 | 1.78 | 1.21, 2.61 | 0.004 |
| Race/Ethnicity |  |  |  |  |  |  |
| White, non-Hispanic (ref) | - | - | - | - | - | - |
| Black, non-Hispanic | 1.25 | 0.64, 2.43 | 0.520 | 0.91 | 0.46, 1.82 | 0.790 |
| Other/2+races, non-Hispanic | 0.68 | 0.38, 1.21 | 0.192 | 0.63 | 0.35, 1.13 | 0.120 |
| Hispanic | 1.09 | 0.58, 2.04 | 0.786 | 0.97 | 0.51, 1.85 | 0.925 |
| Household income | 1.10 | 0.99, 1.23 | 0.078 | 1.09 | 0.97, 1.22 | 0.138 |
| Parental status^a^ |  |  |  |  |  |  |
| Non-caregiver (ref) | - | - | - | - | - | - |
| Parent, child/children < 12 years old | 0.10 | 0.66, 1.51 | 0.982 | 1.07 | 0.70, 1.64 | 0.741 |
| Parent, child/children 12-18 years old | 0.63 | 0.36, 1.11 | 0.111 | 0.76 | 0.42, 1.34 | 0.338 |
| Urbanicity of residence |  |  |  |  |  |  |
| Non-urban (ref) | - | - | - | - | - | - |
| Urban |  |  |  | 0.97 | 0.64, 1.49 | 0.901 |
| Political party identification^b^ |  |  |  | 0.84 | 0.77, 0.92 | <0.001 |
| Intercept | 0.01 | 0.04, 0.25 | <0.001 | 0.18 | 0.06, 0.53 | 0.002 |
| χ², log likelihood | 35.55, -370.56 | | | 49.55, -363.56 | | |
| Notes: OR=Odds Ratio; CI=Confidence Interval. CI = confidence interval. Ref=reference group coded 0; ^a^Parents defined as primary caregiver to child/children aged 18 years and younger who were living at home; ^b^1=*Strong Democrat* to 7=*Strong Republican*. | | | | | | |
